# Potential of bacteriophages as disinfectants to control of *Staphylococcus aureus* biofilms

**DOI:** 10.1186/s12866-021-02117-1

**Published:** 2021-02-20

**Authors:** Jun Song, Hongri Ruan, Li Chen, Yuqi Jin, Jiasan Zheng, Rui Wu, Dongbo Sun

**Affiliations:** Heilongjiang Provincial Key Laboratory of Prevention and Control of Bovine Diseases, College of Animal Science and Veterinary Medicine, Heilongjiang Bayi Agricultural University, No. 5 Xinfeng Road, Daqing, 163319 P. R. China

**Keywords:** Bacteriophage, *Staphylococcus aureus*, Biofilm, Disinfectant

## Abstract

**Background:**

*Staphylococcus aureus* is the causative agent of chronic mastitis, and can form a biofilm that is difficult to completely remove once formed. Disinfectants are effective against *S. aureus*, but their activity is easily affected by environmental factors and they are corrosive to equipment and chemically toxic to livestock and humans. Therefore, we investigated the potential utility of a bacteriophage as a narrow-spectrum disinfectant against biofilms formed by *S. aureus*. In this study, we isolated and characterized bacteriophage vB_SauM_SDQ (abbreviated to SDQ) to determine its efficacy in removing *S. aureus* biofilms.

**Results:**

SDQ belongs to the family *Myoviridae* and consists of a hexagonal head, long neck, and short tail. This phage can sterilize a 10^9^ CFU/mL culture of *S. aureus* in 12 h and multiply itself 1000-fold in that time. Biofilms formed on polystyrene, milk, and mammary-gland tissue were significantly reduced after SDQ treatment. Fluorescence microscopy and scanning electron microscopy showed that SDQ destroyed the biofilm structure. Moreover, the titer of SDQ remained relatively high after the lysis of the bacteria and the removal of the biofilm, exerting a continuous bacteriostatic effect. SDQ also retained its full activity under conditions that mimic common environments, i.e., in the presence of nonionic detergents, tap water, or organic materials. A nonionic detergent (Triton X-100) enhanced the removal of biofilm by SDQ.

**Conclusions:**

Our results suggest that SDQ, a specific lytic *S. aureus* phage, can be used to control biofilm infections. SDQ maintains its full activity in the presence of nonionic detergents, tap water, metal chelators, and organic materials, and can be used in combination with detergents. We propose this phage as a narrow-spectrum disinfectant against *S. aureus*, to augment or supplement the use of broad-spectrum disinfectants in the prevention and control of the mastitis and dairy industry contamination caused by *S. aureus*.

**Supplementary Information:**

The online version contains supplementary material available at 10.1186/s12866-021-02117-1.

## Background

Mastitis, a persistent inflammatory reaction in the udder tissue of dairy cattle, is one of the most important diseases throughout the world [1]. Mastitis not only affects the health of milk-producing animals, with severe financial losses for dairy farmers, but also affects animal welfare [2]. Bovine intramammary infections can be caused by a wide variety of bacteria, fungi, and mycoplasmas [3, 4]. *Staphylococcus aureus* is one of the major pathogens causing bovine mastitis, and seriously affects the health and milk quality of dairy cows [5]. Biofilms play an important role in the spread of *S. aureus* and the persistence of infections [6, 7]. A biofilm is a reticulated polymeric matrix produced by bacteria that can wrap the bacterial cells, and greatly enhances the bacterium’s resistance to the external environment. Several studies have shown that biofilm bacteria are up to 1000-times more resistant to antibiotics than planktonic bacteria [8]. Therefore, it is necessary to prevent and control both *S. aureus* colonization and biofilm formation.

Bovine mastitis prevention strategies, including good infection control and hygiene practices, vaccination, the culling of infected cows, and the cleaning of equipment, reduce the risk of bacterial infection and transmission [9]. It is extremely important to disinfect and sanitize dairy farms. In general, the commonly used broad-spectrum disinfectants belong to chemical categories that have been shown to be flammable, light sensitive, corrosive to metals, irritating, carcinogenic, and/or toxic to livestock and humans [10, 11]. Importantly, several studies have shown that chemical disinfectants can select for mutant bacteria, increasing the risk of the emergence of resistant strains [12, 13]. Tap water, organic materials, or detergents can also reduce the efficacy of chemical disinfectants. Therefore, a new disinfectant is required that can be used in combination with detergents with no diminution of its effectiveness.

Since their discovery in 1915, bacteriophages have been used extensively in human and veterinary medicine, the food industry, and various agricultural settings. They are mainly used to control common bacterial contaminants, such as *Staphylococcus aureus*, *Pseudomonas aeruginosa*, and *Listeria monocytogenes* [14].. As biocontrol agents, phages have the following advantages over conventional antibiotics: high specificity, self-replication, self-limitation, continuous adaption to altered host systems, low inherent toxicity, and easy isolation. Phages have also been shown to effectively remove bacterial biofilms, including those of *S. aureus*, *Streptococcus agalactiae*, *Escherichia coli*, and other major pathogens that cause dairy cow mastitis [15–17]. In this study, a biofilm model was established in mammary-gland tissue to evaluate the removal of biofilms by SDQ [18]. We also investigated the stability and bactericidal activity of SDQ in detergents and other agents. The objective of this study were to investigate the potential utility of SDQ as a disinfectant to help control the mastitis and food contamination caused by *S. aureus*, to lay the foundation for the development of phage-based narrow-spectrum biological disinfectants.

## Results

### Morphology and plaques of SDQ

**Fig. 1:**
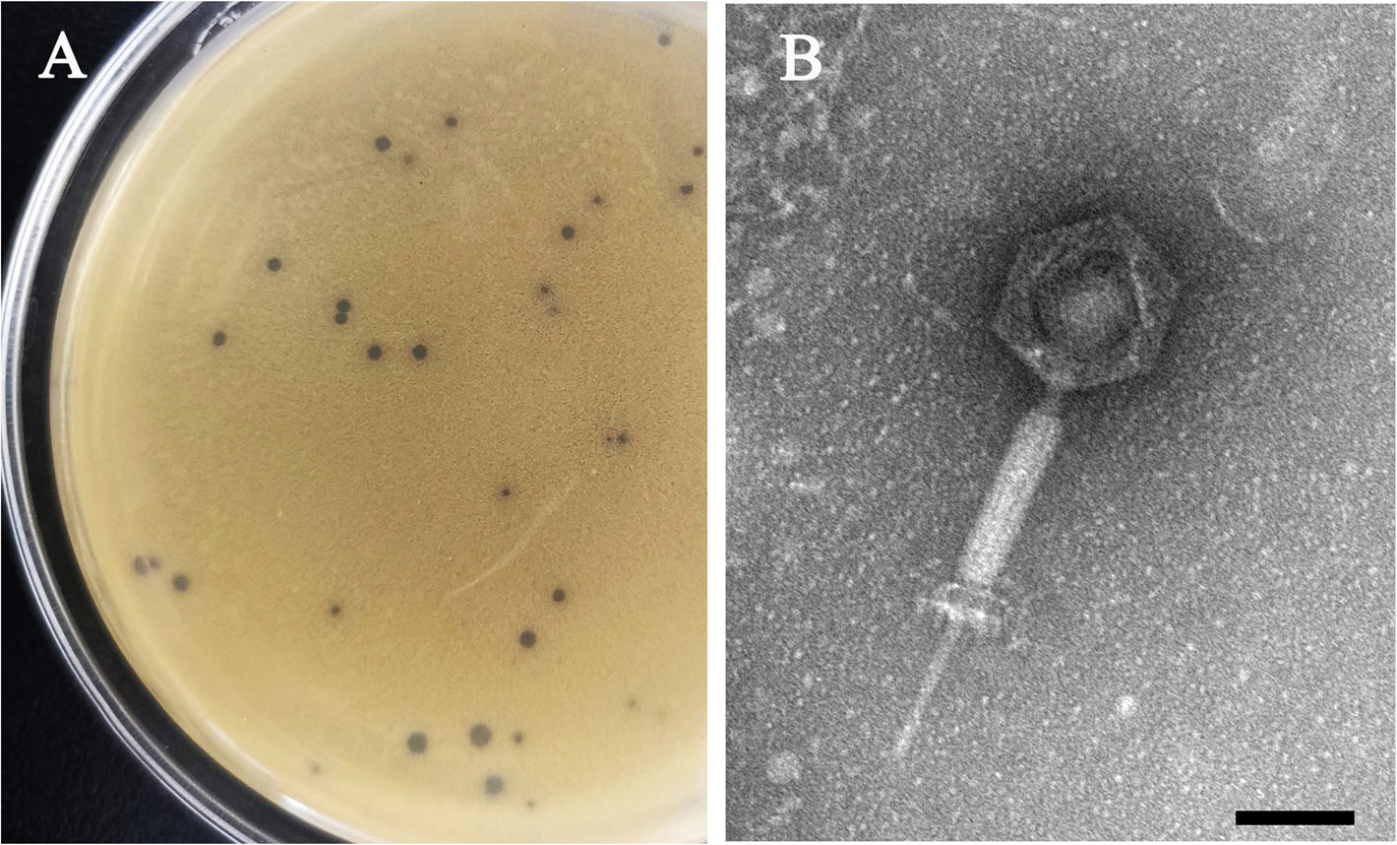
Morphology and plaques of SDQ. **a** Phagocytic plaque of SDQ. **b** SDQ was negatively stained with 2% phosphotungstic acid and examined with transmission electron microscopy at an accelerating voltage of 120 kV. The scale bar represents 50 nm

A *S. aureus* phage, designated vB_SauM_SDQ (abbreviated as SDQ), was isolated from sewage using *S. aureus* ATCC 43300 as the host strain. The plaque of SDQ is shown in Fig. 1a. Light-colored halo rings occur around the phage plaque, indicating that SDQ may secrete polysaccharide depolymerase to enhance its ability to lyse bacteria. Electron micrographic images of SDQ indicated that it has an isometric, icosahedral head, a long neck, and a contractile tail, suggesting that SDQ is a member of the family *Myoviridae*, as shown in Fig. 1b. The diameter of the SDQ head is approximately 75 ± 3 nm (*n* = 3), and the tail length is approximately 189 ± 5 nm (n = 3).

### One-step growth curve

**Fig. 2:**
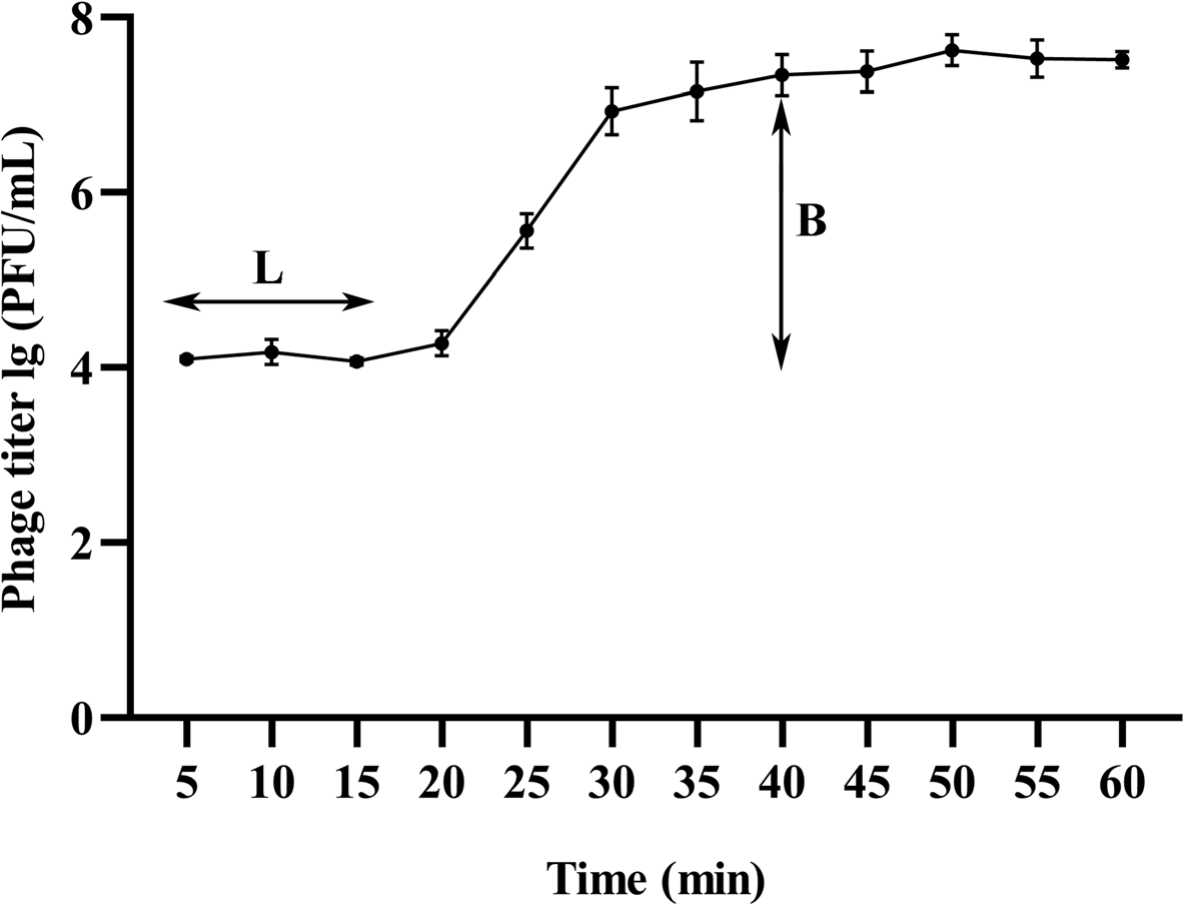
One-step growth curve of SDQ. One-step phage growth of SDQ at 37 °C was investigated. The phage growth parameters are indicated in the figure and correspond to: L, latent period and B, burst size. Data are presented as mean ± SD of three independent experiments

The optimal multiplicity of infection (MOI) determination (Table 1) showed that MOI = 0.01 produced the highest phage production rate. Therefore, when constructing the one-step growth curve, the rates were determined at MOI = 0.01. The one-step growth curve of SDQ infecting *S. aureus* ATCC 43300 revealed an eclipse period of 15 min, a latent phase of 25 min, and a burst size of ~ 220 plaque-forming units (PFU) per infected cell ([2.2 × 10^7^ PFU/mL × 10 mL]/[10^5^ CFU/mL × 10 mL]), indicating that SDQ is an efficient lytic phage (Fig. 2).

**Table 1:** Determination of optimal multiplicity of infection (MOI)

No.	Number of bacteria	Number of phages	MOI	Phage titers/(PFU/mL)
1	10^6^	10^8^	100	1.2 × 10^8^ ± 0.5 × 10^8^
2	10^6^	10^7^	10	2.5 × 10^8^ ± 0.5 × 10^8^
3	10^6^	10^6^	1	4.3 × 10^8^ ± 1.0 × 10^8^
4	10^6^	10^5^	0.1	2.3 × 10^9^ ± 0.7 × 10^9^
5	10^6^	10^4^	0.01	1.7 × 10^10^ ± 0.2 × 10^10^
6	10^6^	10^3^	0.001	5.4 × 10^9^ ± 0.9 × 10^9^

MOI The values indicate means and standard deviations (SD) (*n* = 3)

### pH and thermal stability

**Fig. 3:**
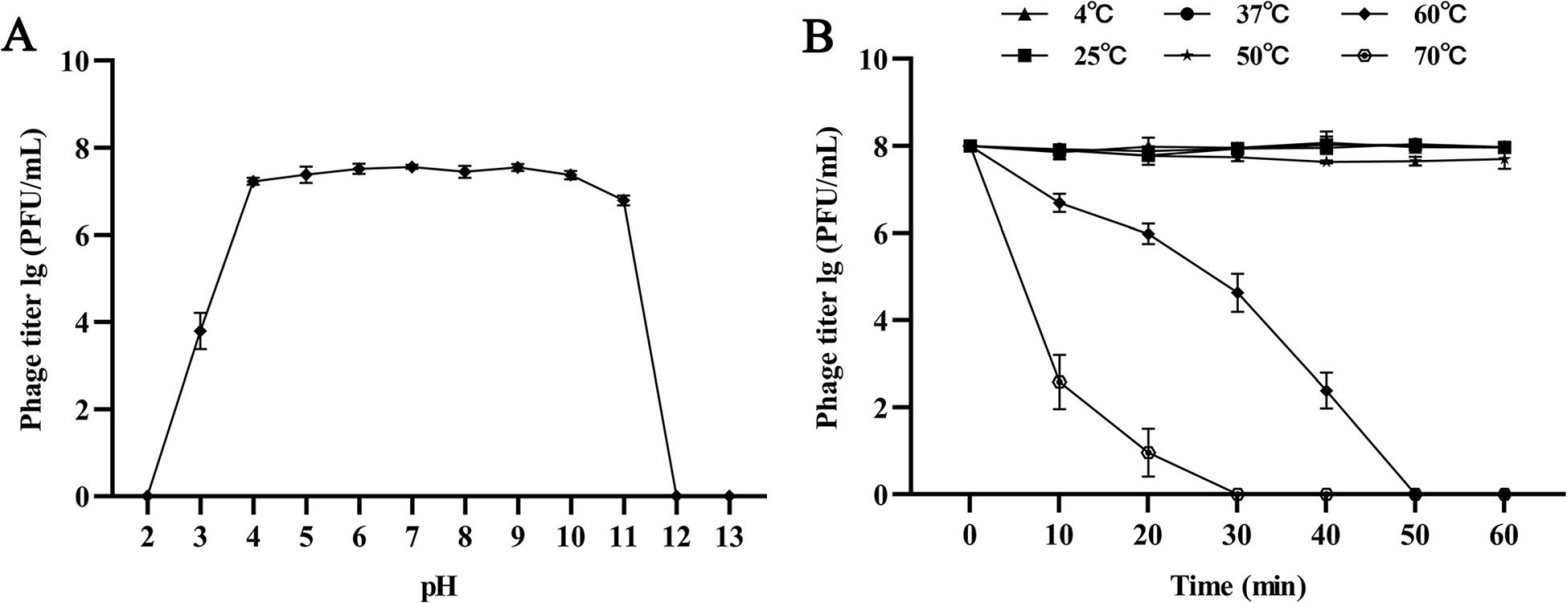
Stability of SDQ. **a** pH stability: SDQ was incubated at different pHs for 1 h. **b** Thermal stability: SDQ was incubated at different temperatures and its activity measured every 10 min. Data are presented as mean ± SD of three independent experiments

pH stability tests showed that SDQ is stable at pH 4.0–11.0. At pH 3.0, SDQ activity decreased significantly, and at pH 12.0 and pH 13.0, no plaque formation was observed (Fig. 3a). SDQ showed 100% stability at room temperature (25 °C) and refrigeration temperature (4 °C), and also good stability after incubation at 37 °C and 50 °C for 1 h. Its activity gradually decreased at 60 °C and it was totally inactive at ≥70 °C (Fig. 3b). SDQ also showed activity after storage at 4 °C for 6 months (Additional file 1: Table S1).

### Host range of SDQ

To determine the host range of SDQ, 26 bacterial strains were used in plaque assays. SDQ showed lytic activity against most *S. aureus* strains, whereas no activity was observed against the other species tested in this study (Table 2 and Additional file 2: Table S2). These results indicate that SDQ is *S. aureus*-specific, with a broad lytic spectrum.

**Table 2:** Host range of SDQ against different clinical isolates of *S. aureus*

Bacterial species	Strain	Phage Sensitivity	Strain Resource	MLST (ST)
*S. aureus*	ATCC 43300	**+**	ATCC	–
	ATCC 29213	**+**	ATCC	–
	ATCC 25923	**+**	ATCC	–
	SA 2Y3–4	**+**	Clinical isolate	97
	SA 2Y9–1	**+**	Clinical isolate	97
	SA 2Y7–1	**+**	Clinical isolate	5817
	SA 2Y4–3	**+**	Clinical isolate	239
	SA 7–1	**–**	Clinical isolate	398
	SA 7–3	**+**	Clinical isolate	398
	SA 8–1	**–**	Clinical isolate	1
	SA 8–2	**–**	Clinical isolate	2154
	SA 8–3	**–**	Clinical isolate	9
	SA 25–4	**+**	Clinical isolate	239
	SA 25–5	**+**	Clinical isolate	97
	SA 27–2	**+**	Clinical isolate	5796
	SA 4-2p	**+**	Clinical isolate	5
	SA-11-2-2p	**+**	Clinical isolate	398
	SA11–1	**+**	Clinical isolate	97
	SA11–2	**+**	Clinical isolate	97
	SA16b	**+**	Clinical isolate	398
*S. agalactiae*	ATCC 13813	**–**	ATCC	–
*L. monocytogenes*	ATCC 19115	**–**	ATCC	–
*E. faecalis*	ATCC 29212	**–**	ATCC	–
*S. Typhimurium*	ATCC 14028	**–**	ATCC	–
*P. mirabilis*	CMCC 49005	**–**	CMCC	–
*E. coli*	ATCC 25922	**–**	ATCC	–

(+) = lytic; (−) = non lytic

### Lytic efficiency of SDQ against planktonic bacteria

**Fig. 4:**
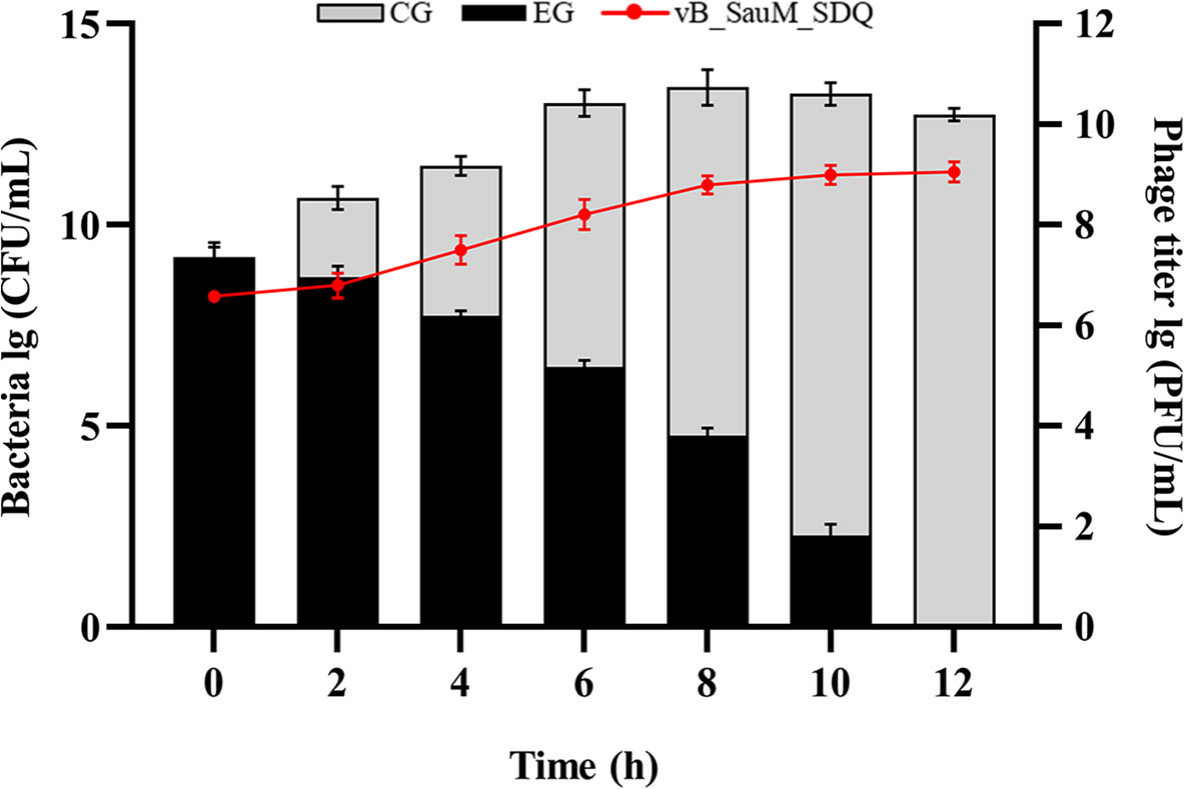
Activity of SDQ against *S. aureus*. The Black histogram shows the removal of bacteria by SDQ (experimental group, EG); the gray histogram shows the changes in phage-untreated bacteria (control group, CG); line graph shows the changes in the SDQ titer. Data are presented as mean ± SD of three independent experiments

The ability of SDQ to remove the planktonic form of methicillin-resistant *S. aureus* (MRSA) 25–4 was evaluated at various time points after its introduction (2, 4, 6, 8, 10, and 12 h). The number of planktonic bacteria decreased with time, as shown in Fig. 4. Compared with the control group, the number of bacteria had decreased by 99% after 10 h. After SDQ treatment for 12 h, the bacterial suspension was clear, with a large amount of bacterial debris, and no viable bacteria were detected. The titer of SDQ increased by three orders of magnitude during this period.

### Biofilm sensitivity to SDQ

**Fig. 5:**
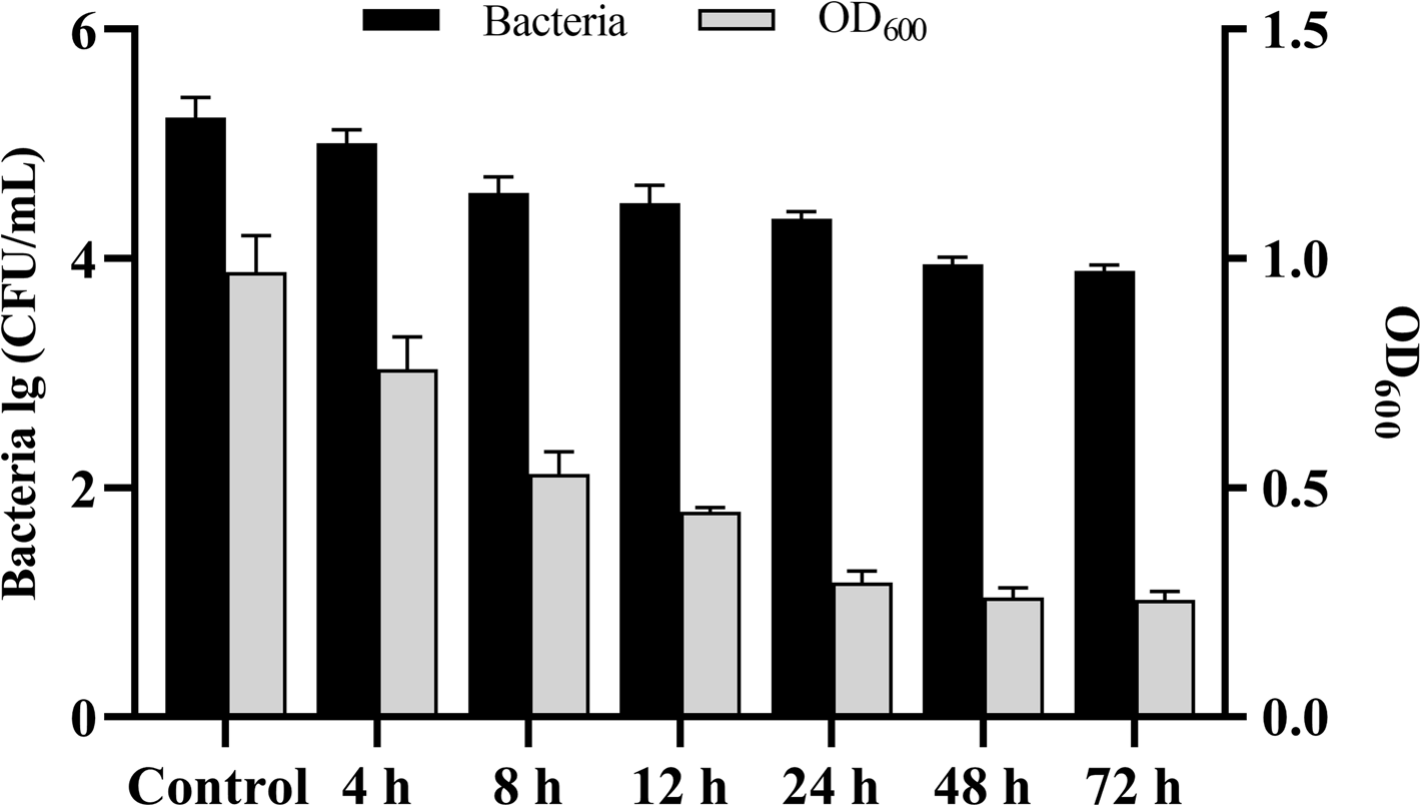
Reduction in established *S. aureus* biofilm after challenge with SDQ for 4, 8, 12, 24, 48, or 72 h. The black histogram shows the removal of bacteria in the biofilm by SDQ; the gray histogram shows the removal of biofilm by SDQ. Data are presented as mean ± SD of three independent experiments

SDQ showed strong antibiofilm activity in the process of destroying biofilms (Fig. 5). In the control group, the concentration of SA 25–4 in the biofilm was 1.89 × 10^5^ CFU/mL, but as the SDQ treatment time increased, the number of bacteria in the biofilm decreased. Compared with the control group, the bacterial concentration in the biofilm decreased by 9.54 × 10^4^ CFU/mL (50%), 1.67 × 10^5^ CFU/mL (88%), and 1.81 × 10^5^ CFU/mL (95%) at 4, 24, and 48 h, respectively, indicating that SDQ effectively removed the biofilm. After crystal violet staining, the measured absorbance OD_600_ was reduced from 0.98 to 0.25, confirming that SDQ effectively removed the biofilm. SDQ not only effectively removed an already-formed biofilm, but also prevented the formation of a bacterial biofilm (Additional file 3: Table S3). Therefore, SDQ both effectively prevents and removes bacterial biofilms.

**Fig. 6:**
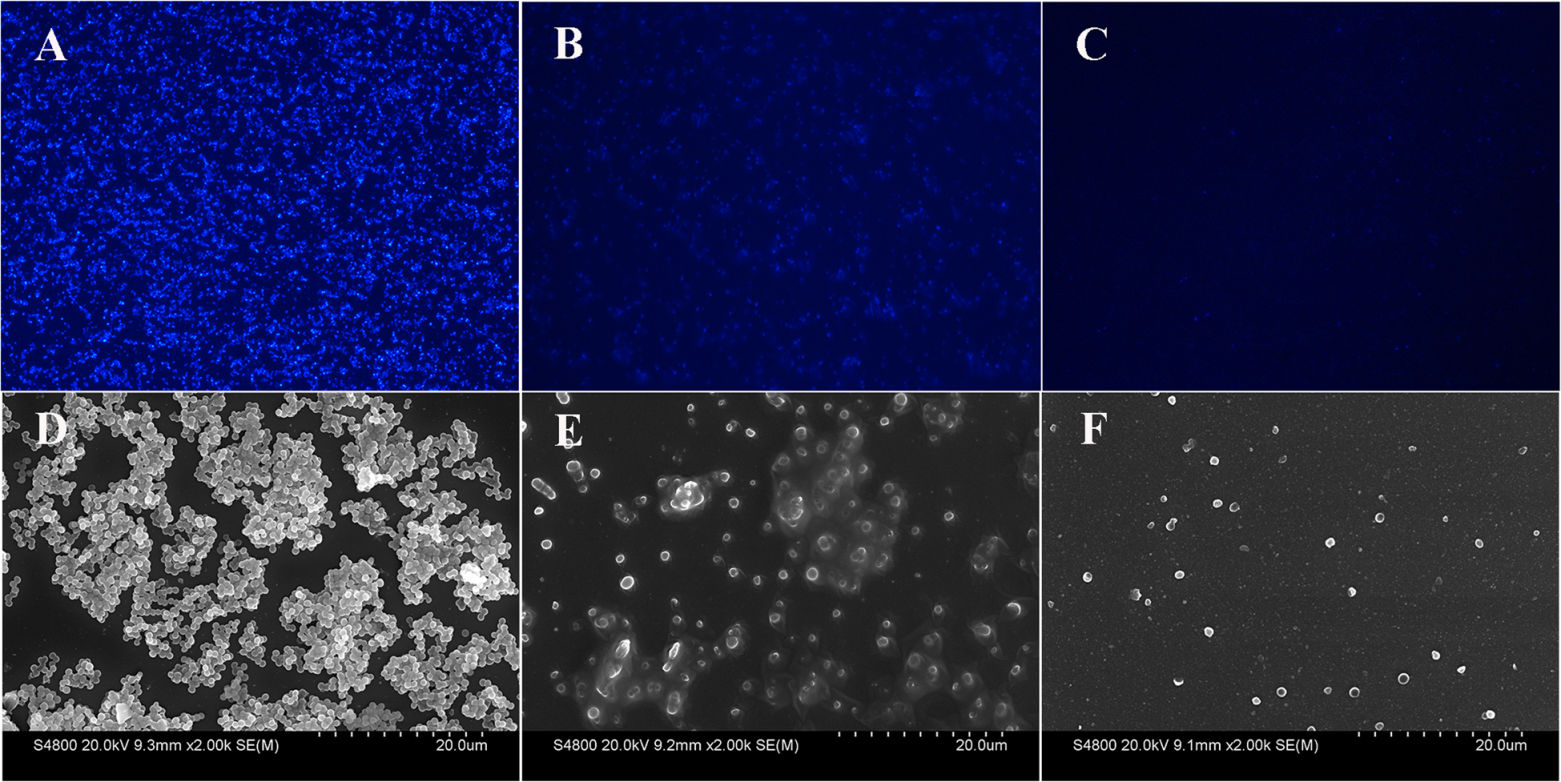
Observation of biofilms with fluorescence microscopy and SEM. **a**, **b**, **c** Fluorescence microscopic observations at 0, 24, and 48 h, respectively (200×). D, E, F SEM observations at 0, 24, and 48 h, respectively. The numbers of bacteria in the biofilms were significantly reduced at 24 h and 48 h after SDQ treatment

Fluorescence microscopy and scanning electron microscopy (SEM) observations showed that SA 25–4 formed a dense biofilm structure on glass coverslips (Fig. 6a, d). After SDQ treatment for 24 h, the bacterial density in the biofilm had decreased significantly (Fig. 6b, e). After SDQ treatment for 48 h, the integrity of the bacterial biofilm structure was destroyed (Fig. 6c, f), indicating that SDQ has a strong capacity to kill bacteria and destroy the biofilm structure.

### Removal of biofilm from milk by SDQ

**Fig. 7:**
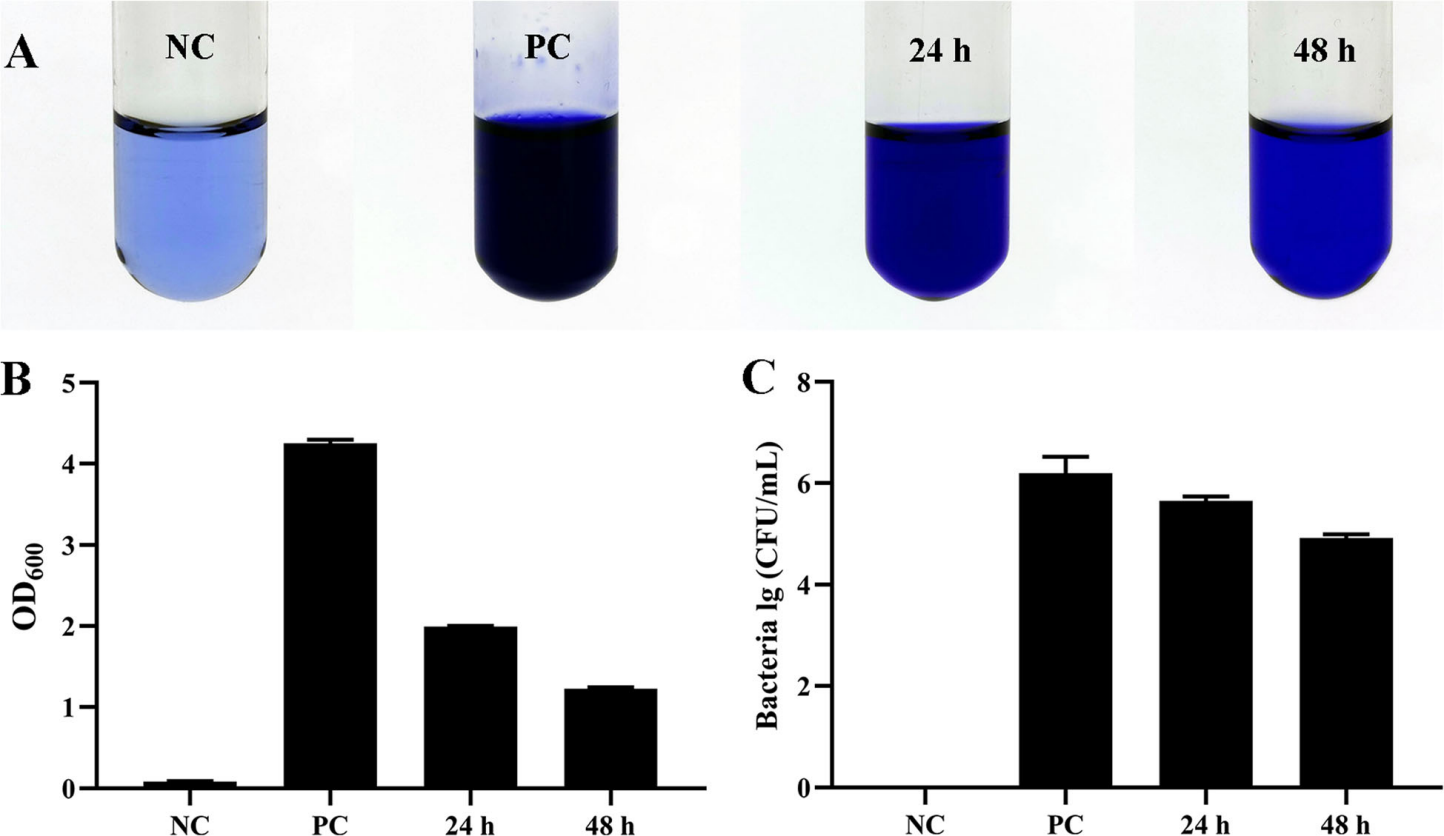
Removal of biofilm from milk by SDQ. **a** Biofilm staining assay demonstrated the disruption of biofilms incubated with SDQ for 24 or 48 h. The assay was performed in triplicate in glass test tubes. **b** Optical density at 600 nm (OD_600_) is represented in the graph as mean ± SD values. **c** Viable-cell plate counting assay demonstrates the removal of cells from biofilms after SDQ treatment for 24 and 48 h. Bar chart shows CFU/mL

SDQ showed strong antibiofilm activity when destroying biofilms in milk (Fig. 7). Crystal violet staining showed that after treatment with SDQ for 24 h, the OD_600_ decreased from 4.21 to 1.95 (by 54% compared with the positive control group), and at 72 h, the OD_600_ had decreased by 3.02 units (71%) (Fig. 7a, b), indicating that SDQ effectively removed the biofilm from milk. In the positive control group, the concentration of SA 25–4 in the biofilm was 1.57 × 10^6^ CFU/mL, but as the SDQ treatment time increased, the number of bacteria in the biofilm decreased. Compared with the positive control group, the bacterial concentration in the biofilm at 24 h had decreased by 1.09 × 10^6^ CFU/mL (69%), and at 48 h, the bacterial concentration had decreased by 1.48 × 10^6^ CFU/mL (95%) (Fig. 7c), confirming that SDQ effectively removed the biofilm from milk.

### SDQ treatment of *S. aureus* biofilm formed in mammary-gland tissue

**Fig. 8:**
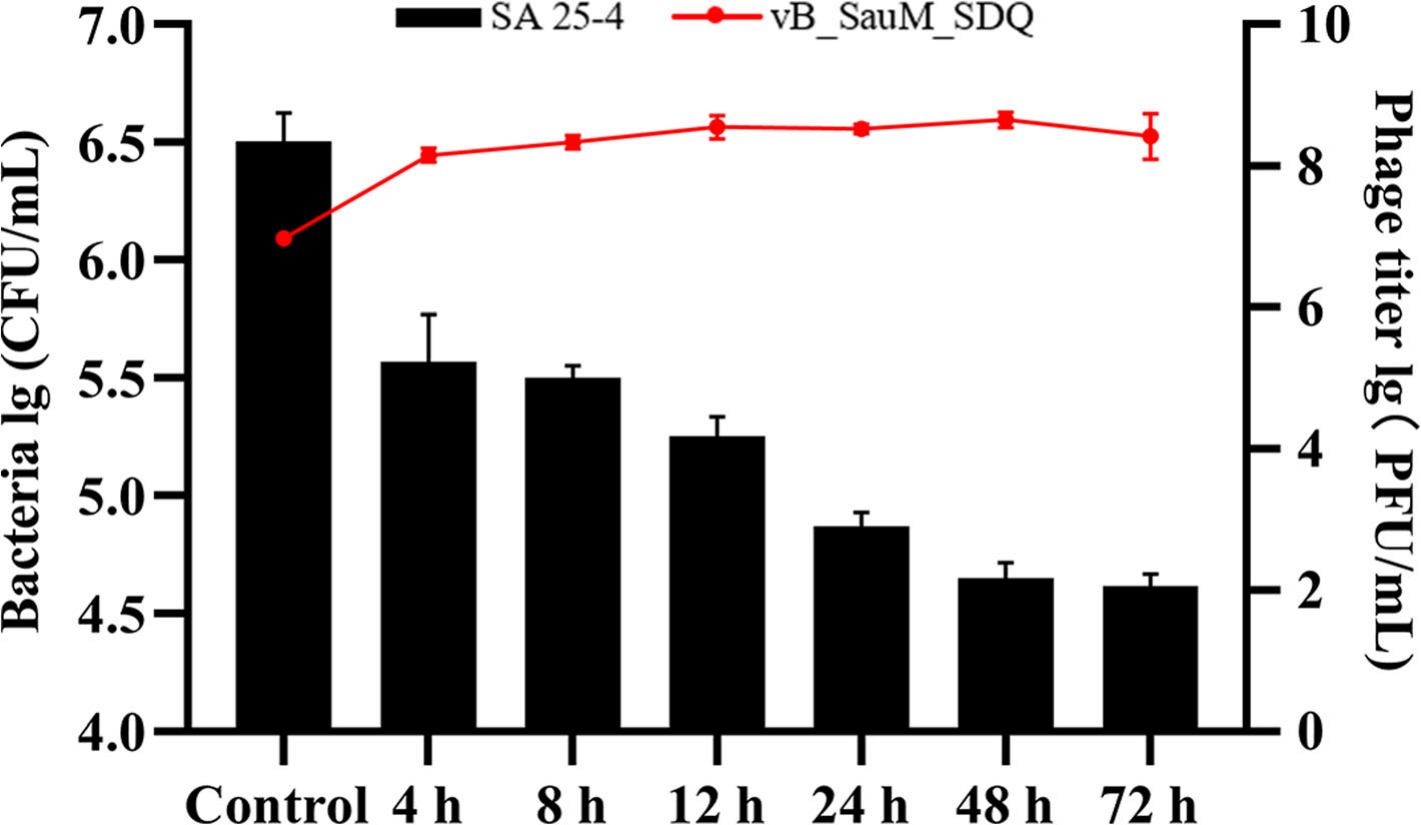
Removal of biofilm from mammary-gland tissue by SDQ. The histogram shows the removal of bacteria from the biofilm by SDQ; line graph shows the changes in the SDQ titer. Data are presented as mean ± SD of three independent experiments

SDQ not only destroyed the biofilm in mammary-gland tissue, but also prevented its formation, thus showing strong antibiofilm activity (Fig. 8). After 72 h, the bacterial concentration had decreased from 3.28 × 10^6^ CFU/mL to 3.95 × 10^4^ CFU/mL, and the number of bacteria in the biofilm had decreased by 98%, indicating the clear removal of the biofilm, and the concentration of SDQ increased from 9.15 × 10^6^ PFU/mL to 3.52 × 10^8^ PFU/mL. Therefore, the maintenance of a high phage concentration contributed to the continuous removal of the biofilm.

### Effects of detergents and environmental factors on SDQ

To determine whether detergents affect the bactericidal efficacy of SDQ, the phage was tested in combination with detergents or under the harsh conditions that might occur during its application. SDQ was mixed with SA 25–4 in the presence of various compounds, and the biofilm was monitored with the plaque assay to determine the lytic activity of SDQ relative to the control. If the detergents did not inactivate the phage, they were expected to eliminate the biofilm because they disperse organic matter from surfaces. Various detergents (1% final concentration) were used with the phage to determine whether their addition improved the bactericidal efficacy of SDQ.

**Fig. 9:**
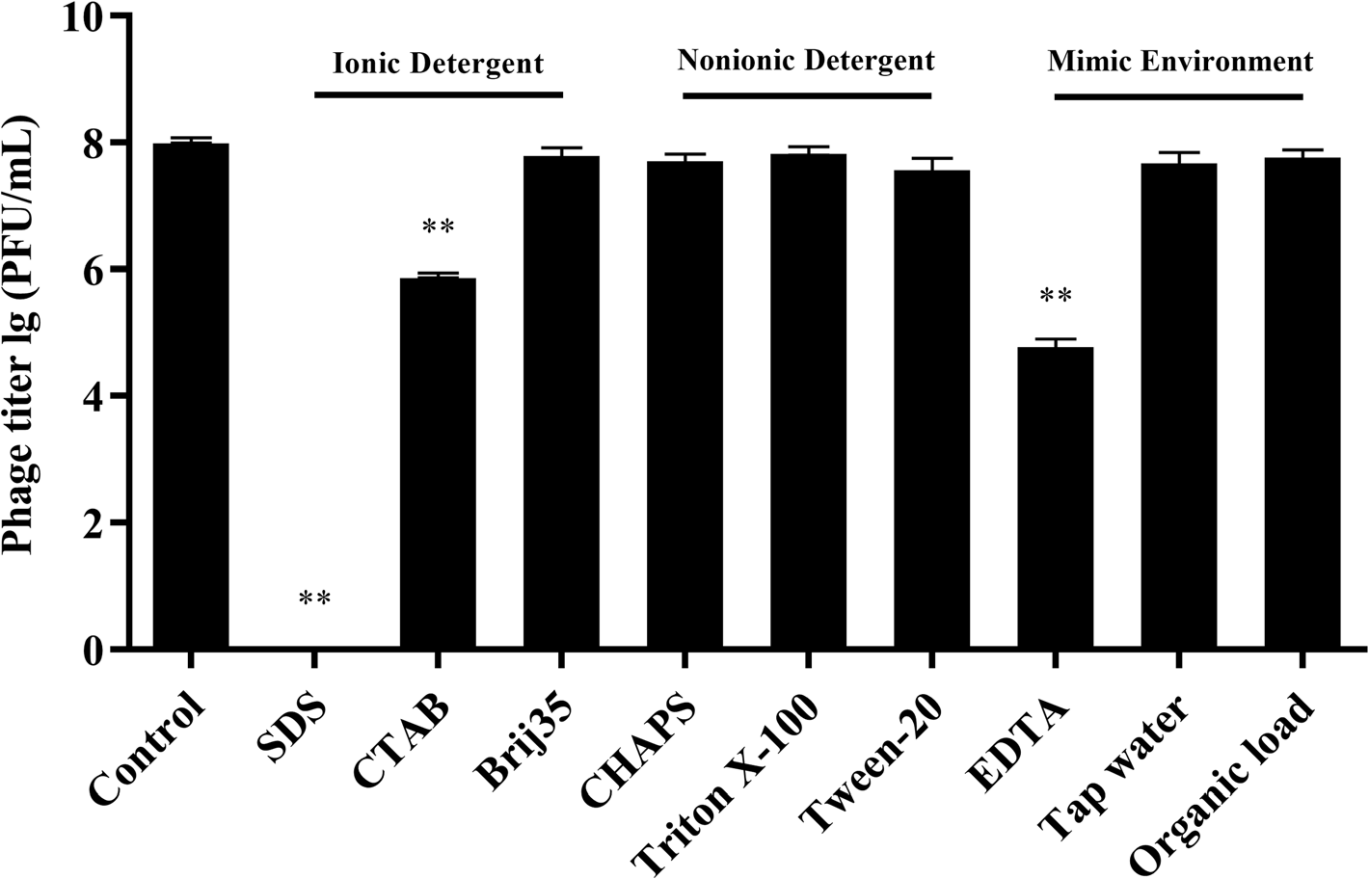
SDQ activity in the presence of detergents and environmental factors. SDQ was tested against *S. aureus* in the presence of 1% detergent (SDS [anionic], CTAB [cationic], CHAPS [zwitterionic], Triton X-100, Tween 20, and Brij 35 [all nonionic]), 10 mM EDTA, tap water, or 10% organic material (fetal bovine serum) with the plaque assay. Data are presented as mean ± SD of three independent experiments

Notably, as shown in Fig. 9, an anionic detergent (sodium dodecyl sulfate, SDS) inactivated SDQ, whereas hexadecyl trimethyl ammonium bromide (CTAB) and ethylenediaminetetraacetic acid (EDTA) significantly reduced the activity of SDQ. However, SDQ retained its full activity in zwitterionic (CHAPS) and nonionic detergents (Triton X-100, Tween 20, and Brij 35). SDQ was also tested in 10% fetal bovine serum, which represents an organic soil load, as defined by the Association of Official Analytical Chemists (AOAC) [19]. Under organic load conditions, SDQ was as active as the control. SDQ was then dialyzed against distilled water and tested in the absence of any buffer. Compared with the control, SDQ retained its full activity.

**Fig. 10:**
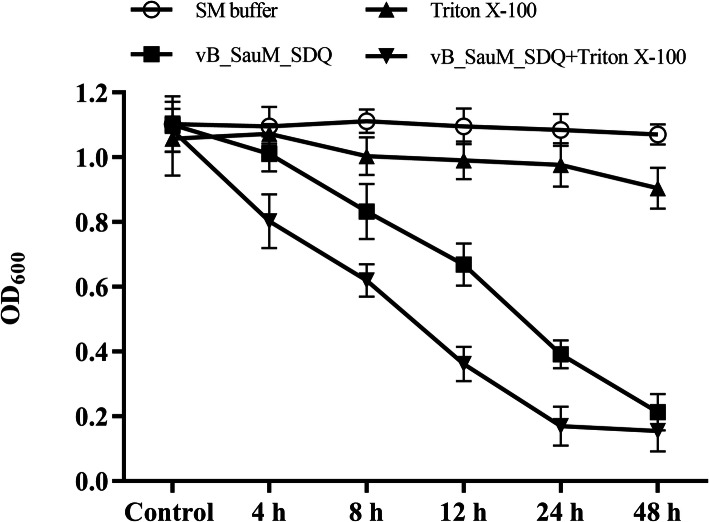
Synergistic effects of SDQ and detergent on biofilm. At the indicated times, the numbers of cells in biofilm treated with SM buffer (black circles), vB_SauM_SDQ (black squares), Triton X-100 (black triangles), or vB_SauM_SDQ + Triton X-100 (black inverted triangles) were determined by the optical density at 600 nm. Data are presented as mean ± SD of three independent experiments

To identify the synergistic effects of SDQ and detergents, established biofilms were treated with SDQ, Triton X-100, or a combination of SDQ and Triton X-100. As shown in Fig. 10, compared with SDQ, Triton X-100 + SDQ significantly reduced the mass of the SA 25–4 biofilm within 12 h.

## Discussion

*Staphylococcus aureus* is one of the commonest causative agents of chronic mastitis. It can form biofilms and is therefore difficult to completely remove once established. Dairy cow mastitis caused by *S. aureus* does not respond well to antibiotic therapy, and infected cows must be segregated or culled from the herd. Therefore, effective preventive measures to eliminate the source of infection are very important [9]. Bacteriophage prophylaxis is a possible alternative strategy to achieve this goal. We studied the biological characteristics of SDQ and analyzed its ability to prevent and control biofilm formation. Our results showed that SDQ effectively prevented and controlled planktonic *S. aureus* bacteria and *S. aureus* biofilms. Therefore, it has potential utility as a disinfectant to control the mastitis and food contamination caused by *S. aureus*.

SDQ has been identified as a virulent phage with strong lytic efficacy against MRSA. It can reduce the MRSA concentration in culture medium to below the detection limit within 12 h, indicating that it has potentially applicable in the treatment of clinical infections. However, the thermal stability and acid-base properties are the foundations of phage use in the clinical context [20]. In this study, SDQ was stable at < 50 °C and within the range of conventional pHs (4–11). The temperature of the dairy cow feeding environment usually does not exceed 24 °C, whereas the normal temperature range of dairy cows is 37.5–39.5 °C, so it is unlikely that SDQ will lose its activity in response to excessive temperature variations during its use. The pH range of fresh milk is 6.4–6.6, indicating that SDQ should be stable in milk. Furthermore, the titer of SDQ remains stable for more than 6 months at 4 °C, which is convenient for storage. SDQ also multiplies during its sterilization of *S. aureus*. Phage self-replication is an important parameter that allows phages to be used as both antibacterial agents and protective or preservative agents in food to control the growth of foodborne pathogens in the food industry. Bacteriophage products are already in use in agricultural, food safety, and diagnostic applications, confirming the utility and viability of this approach. In this study, bacteriophage SDQ showed lytic activity against both *S. aureus* clonal complexes CC97 and CC398, and part of clonal complex CC1 strain (Additional file 2: Table S2). In the treatment of anti-phage bacterial strains and drug-resistant bacterial strains, phage can be used in combination with antibiotics, antimicrobial peptides, and other antibacterial drugs or in a phage cocktail to control resistant bacterial strains. *Staphylococcus aureus* SA 25–4 belongs to the CC97 strain, which is one of the main branches of ruminant-adapted *S. aureus*, so the results presented here are representative. All its characteristics strongly suggest that SDQ is a potential preventative and/or therapeutic bacteriophage that can be used against MRSA infections in ruminants.

Most infections are associated with biofilm formation [8]. Biofilms are recognized as a significant problem in many industries, including the medical and food industries, and also pose a threat to the environment [21–23]. Biofilm-forming *S. aureus* strains are generally much more difficult to kill and are more resistant to antimicrobial agents than their planktonic counterparts [24]. Previous studies have demonstrated that phages can potentially remove and/or disrupt *S. aureus* biofilms. Morris et al. showed that phages are more effective than antibiotics in biofilm removal [25, 26], and Alves et al. demonstrated that phage K disrupts the integrity of biofilms [27]. In this study, we evaluated the ability of SDQ to remove biofilm from polystyrene plastic plates, glass coverslips, milk, and mammary tissues. Bovine mammary tissue contains many mammary ducts and mammary alveoli. The biofilms on mammary tissue are more complex than those on polystyrene or glass and are therefore closer to the actual situation in cows. Therefore, in this experiment, we used the method of the Milho et al. (2019) to establish an in vitro model of isolated mammary-gland tissue in which to evaluate the effect of SDQ on the *S. aureus* biofilm [18]. The number of bacteria in the biofilm decreased significantly after SDQ treatment. Fluorescence microscopy and SEM showed that the integrity of the biofilm structure was destroyed after SDQ treatment. SDQ also removed *S. aureus* biofilm from milk. In summary, SDQ effectively removed biofilms from polystyrene, glass, milk, and mammary-gland tissues. Consistent with previous research results, our findings show that SDQ has potential utility in the prevention and treatment of bovine mastitis [28, 29]. SDQ formed plaques surrounded by a halo, indicating that this phage expresses polysaccharide depolymerase, an enzyme that may enhance the antibiofilm activity of phages [30, 31]. Although SDQ does not have the broad-spectrum bacteriocidal activity common to most disinfectants, it has advantages that make it superior to common disinfectants in this context. The bacteriophage is nontoxic to livestock or humans and is environmentally safe, noncorrosive, economic, and easy to obtain. Therefore, SDQ can be used as a “biological disinfectant” or environmental bactericide capable of controlling *S. aureus* biofilms.

However, inhibitors maybe encountered during its practical application, such as detergents, organic material, etc. Disinfectants are often used in conjunction with detergents. Detergents permeate, disperse, and remove smudges from surfaces, thereby allowing disinfectants to work. However, the activity of disinfectants is often reduced or they are inactivated by detergents [32]. In this study, we examined the effect of detergents on the bactericidal efficacy of SDQ. Ionic detergents reduced the activity or inactivated SDQ, but these are seldom used as cleaning agents, whereas SDQ maintained full bacteriolytic activity in the presence of nonionic detergents (Triton X-100, Tween 20, and Brij 35). In practice, nonionic detergents have good emulsification, penetration, and dispersion properties, rendering them effective detergents [33–35]. Therefore, these detergents can assist SDQ to disrupt the *S. aureus* biofilms.

According to the AOAC, 10% fetal bovine serum represents an organic soil load [19]. To test the practical effectiveness of SDQ, it was diluted with tap water and tested in the absence of any buffer. Compared with the control, the full activity of SDQ was retained in both tap water and fetal bovine serum. In some region, problems such as hard water might be encountered, which can bind to either disinfectants or detergents, interfering with their effectiveness [36]. Metal chelators, such as EDTA, are commonly used to treat hard water. However, the addition of 10 mM EDTA did not reduce the lytic activity of SDQ for *S. aureus* cells. Therefore, the use of this phage combined with a nonionic detergent can better eliminate a biofilm than the biofilm alone and is unaffected by organic matter or tap water.

## Conclusions

In this study, SDQ not only prevented and removed *S. aureus* biofilms, but also multiplied during the process of infection, making it a good candidate for further preventative and therapeutic development. SDQ also retained its full activity in the presence of nonionic detergents, tap water, a metal chelator, and organic material, and can be used in combination with detergents. Although bacteriophage will never replace traditional disinfectants, they have great potential utility as narrow-spectrum biological disinfectants for controlling *S. aureus* infections and the devastating effects of MRSA and related biofilms, such as occur on medical equipment and in the food industry and livestock farming.

## Methods

### Bacterial strains and culture conditions

A selection of 26 different bacterial strains, including *S. aureus, Streptococcus agalactiae, Enterobacter faecalis, Salmonella typhimurium, Proteus mirabilis, Listeria monocytogenes*, and *E. coli*, were purchased from the American Type Culture Collection (ATCC, Manassas, VA, USA) and the National Center for Medical Culture Collections (CMCC, Beijing, China), and 17 clinical *S. aureus* isolates were provided by Heilongjiang Provincial Key Laboratory for the Prevention and Control of Bovine Diseases (Daqing, Heilongjiang, China). These clinical strains were tested with PCR amplification of the *mecA* gene to confirm that the isolates were MRSA. We identified the multilocus sequence typing (MLST) types of these strains, and the 17 clinical strains of *S. aureus* included nine ST types (Additional file 2: Table S2). All the strains were streaked on tryptic soy agar (Hopebio, Qingdao, China) before experimentation, and single colonies were recovered by culture in tryptic soy broth (Hopebio) overnight at 37 °C to ensure the purity of the bacterial stocks. All bacterial strains were preserved in 20% (v/v) glycerol and the stocks were maintained at − 80 °C.

### Biological characteristics of the phage

#### Isolation of the *S. aureus* phage

Phage SDQ was isolated from a dairy farm sewage system using *S. aureus* subsp. aureus Rosenbach (ATCC 43300) as the host strain. Sewage samples were collected from a cattle farm sewer system (Daqing, Heilongjiang, China), centrifuged (9000×g, 4 °C, 10 min), and filtered through 0.22 μm membrane filters (SteriFlip, Millipore). The filtered raw sewage (100 mL) was placed in sterile 100 mL flasks with double-strength TSB. *S. aureus* ATCC 43300 and the prepared sewage samples were then cocultured overnight at 37 °C. The cocultures were centrifuged at 10,000×g for 10 min at 4 °C, and the supernatants were collected and filtered (0.22 μm). A double-layer agar plate assay was used to detect and purify the phage from the supernatant [37]. Briefly, we picked a single spot from a double-layer plate and 100 μL of the host strain and mixed them with 5 mL of molten soft agar (0.75%) medium. This was then overlain on the surface of solidified basal Luria–Bertani (LB) agar. The sample was then incubated for 6–8 h at 37 °C. This was repeated three times to obtain the purified phage lysate. The purified phage was then amplified and stored in dimethyl sulfoxide (DMSO, 3:1 [v/v]) at either 4 °C or − 80 °C.

Following large-scale culture, SDQ was precipitated with 10% (w/v) polyethylene glycol (PEG) 8000 and 1 M NaCl. The phage sample was then placed on the top of a discontinuous CsCl gradient (1.32, 1.45, 1.50, and 1.70 g/mL) and centrifuged at 35,000×g for 3 h at 4 °C. The phage band was collected and dialyzed against a suspension medium (SM) buffer (0.01% gelatin, 100 mM NaCl, 50 mM Tris-HCl, 10 mM MgSO_4_) at 4 °C.

#### Morphology of SDQ

The morphology of SDQ was examined with transmission electron microscopy (TEM; JEM-2100, JEOL, Tokyo, Japan). SDQ was purified and transferred to carbon-coated copper film for 1 min and negatively stained with 2% phosphotungstic acid (pH 7.0). The excess liquid was removed, and the sample air-dried on the carbon-coated copper grid. The stained SDQ was observed under TEM (120 kV).

#### One-step growth

‘MOI’ refers to the ratio of the phage to the host bacteria during the processes of infection [38]. *S. aureus* ATCC 43300 was grown to logarithmic phase and transferred into fresh LB broth at a final concentration of 10^7^ CFU/mL. SDQ was then added at different MOIs (phage/bacteria = 0.001, 0.01, 0.1, 1, 10, or 100) and the mixtures were incubated for 8 h. Immediately after serial dilution, the phage titer in each sample was determined with the double-layer agar plate method.

To construct the one-step growth curve, SDQ was added to an exponential-phase culture of *S. aureus* (1 × 10^5^ CFU/mL) at an MOI of 0.01 and allowed to adsorb for 10 min at 37 °C. The mixture was then centrifuged at 12,000×g for 5 min at 4 °C, and the pellet was resuspended in 10 mL of LB broth. The suspension was then incubated at 37 °C with shaking at 180 rpm. The sample was collected at 5 min intervals until 60 min. The phage titer of the lysates was quantified with the double-layer agar plate method and the growth curve for SDQ was constructed from these data [39]. The experiments were performed in triplicate.

#### pH and thermal stability

To measure the stability characteristics of SDQ, its survival rate was determined after treatment with diverse ranges of pHs and temperatures. Briefly, for the pH stability test, SDQ was incubated at pH 2.0, 3.0, 4.0,5.0, 6.0, 7.0, 8.0, 9.0, 10.0, 11.0, 12.0, and 13.0 for 1 h at 37 °C. For the thermal stability test, SDQ was incubated at 4, 25, 37, 50, 60, and 70 °C, and the phage titers were measured every 10 min. After treatment, all the samples were diluted and tested immediately with the double-layer agar plate method. To test SDQ stability after long-term storage, aliquots of phage suspensions were stored at 4 °C for 6 months. All the experiments were performed in triplicate.

### Antimicrobial activity of SDQ

#### Determination of SDQ the host range

The host range of SDQ was determined with spot tests against a panel of 21 strains [40]. In the spot test experiment, 100 μL droplets of phage stock (10^9^ PFU/mL) were spotted onto freshly seeded lawns of the indicated bacterial strains. The production of lytic spots was assessed after incubation for 12 h at 37 °C. The details of the bacterial strains used in the study are listed in Table 2.

#### Lytic efficiency of SDQ against planktonic bacteria

The experimental strain to be tested (SA 25–4) was grown overnight in LB broth. The overnight bacterial culture (100 μL) was diluted in LB broth to a final titer of approximately 10^9^ CFU/mL. SDQ was added at an MOI of 0.01 and incubated at 37 °C with shaking at 180 rpm in an incubator. The colonies in the culture broth were counted at different time points (0, 2, 4, 6, 8, 10, and 12 h), and a phage-free treatment was used as the control. The bacterial count was calculated from the number of colonies on the plate. The change in phage titer was determined with the double-layer agar plate method [41]. All the experiments were performed in triplicate.

### Antibiofilm activity of SDQ

#### Phage treatment of *S. aureus* biofilm in 96-well cell culture plates

A 96-well cell culture plate was used to detect the inhibitory effect of SDQ on biofilm formation at MOI = 0.01, and to investigate the removal of the biofilm by SDQ. In each well of a 96-well cell culture plate, TSB was inoculated with SA 25–4 at a final concentration of 10^6^ CFU/mL. For the biofilm formation inhibition experiment, SDQ was added to the bacterial culture to a final titer of 10^4^ PFU/mL, and incubated statically at 37 °C for 48 h. For the biofilm removal experiment, SA 25–4 was initially incubated under the conditions described above for 48 h to allow biofilm formation, and then treated with SDQ at a final titer of 10^7^ PFU/mL for 4, 8, 12, 24, 48, or 72 h. A phage-free treatment was used as the control. Viable-cell plate counting was used to determine the numbers of bacteria in the biofilms at different time points, and biofilm removal was measured with crystal violet staining. Each well was rinsed five times with sterile phosphate-buffered saline (PBS) and allowed to air-dry. The SDQ-treated biofilm in each well was stained with 5% crystal violet solution (Becton Dickinson, Sparks, MD) at 25 °C for 60 min, eluted with 33% acetic acid, and the OD_600_ measured with a spectrophotometer (Beijing Purkinje General Instrument Co, Beijing, China) [42]. All the experiments were performed in triplicate.

#### Microscopic imaging of biofilms

The effects of SDQ on the biofilms were assessed with Hoechst 33342 stain (Beyotime Biotechnology, Shanghai, China) and visualized with fluorescence microscopy (Ti2, Nikon Corporation, Japan). Bacterial biofilms were grown on glass coverslips as described above. The nonadherent cells were removed and the wells were washed three times with sterile PBS. The biofilms were treated with SDQ at a final titer of 10^7^ PFU/mL for 0, 24, or 48 h. The glass coverslip was then washed once with sterile PBS. The biofilm was stained with Hoechst 33342 according to the manufacturer’s protocol and visualized with fluorescence microscopy.

For DSEM, the biofilms were cultured as for fluorescence microscopy. The glass coverslips were washed once with sterile PBS. Each biofilm was immobilized with 5% glutaraldehyde, dehydrated a graded series of ethanol concentrations (20, 50, 70, 90, and 100%), and then freeze-dried before SEM analysis (Hitachi S-4800; Hitachi High-Technologies Europe GmbH, Krefeld, Germany).

#### Removal of biofilm from milk by SDQ

To study the removal of biofilm from milk by SDQ, we used pasteurized milk as the culture medium for the *S. aureus* biofilm. In this experiment, a constant temperature oscillator was used to keep the milk in a flowing state while the biofilm was cultured in a glass test tube. A logarithmic-growth-phase culture of SA 25–4 at a final concentration of 10^6^ CFU/mL was added to 1 mL of milk and cultured at 37 ° and 120 rpm for 48 h. The nonadherent cells were removed by washing the biofilm three times with sterile PBS. SDQ at a final titer of 10^7^ PFU/mL was added for 24 or 48 h. A phage-free treatment was used as the positive control, and milk was used as the negative control to compare the removal of the biofilm by the phage. The number of bacteria in the biofilm at different time points was determined with viable-cell plate counting, and biofilm removal was measured with crystal violet staining. Each sample was rinsed five times with sterile PBS and allowed to air-dry. The SDQ-treated biofilms in each sample were stained with 0.5% crystal violet solution (Becton Dickinson) for 60 min at 25 °C and eluted with 33% acetic acid. The OD_600_ of the eluate was measured with a spectrophotometer (Beijing Purkinje General Instrument Co.). All the experiments were performed in triplicate.

#### Phage treatment of *S. aureus* biofilm formed in mammary-gland tissue

The animal trial in this study was approved by the Institutional Animal Care and Use Committee (IACUC) of Heilongjiang Bayi Agricultural University, and conventional animal welfare regulations and standards were followed. A mammary gland from a healthy dairy cow was obtained from a local farm. The mammary gland was immediately sterilized with a previously described method [43]. Briefly, the mammary gland was washed with saline for 10 min, and then placed in alcohol (75%) for 2–3 min. This was repeated twice. The gland was placed in a desiccator for 30 min and washed three times with sterile PBS. Cuboids (10 × 10 × 5 mm^3^) of tissue were cut with a scalpel and frozen at − 20 °C until use. The treated mammary-gland tissue was transferred to a 24-well cell culture plate, and 1.8 mL of TSB medium was added together with 200 μL of 1 × 10^6^ CFU/mL SA 25–4. The samples were incubated at 37 °C for 48 h. To remove the nonadherent cells, the wells were washed five times with sterile PBS. SDQ, at a final titer of 10^7^ PFU/mL, was added for 24, 48, or 72 h, and a phage-free treatment was used as the control group. PBS (10 mL) was then added and the samples shaken vigorously. The change in bacterial titer was determined with the viable-cell plate counting method and the change in phage titer was measured with the double-layer agar plate method. All the experiments were performed in triplicate.

#### Effects of added detergents and environmental factors on SDQ eradication of *S. aureus*

To determine the infectivity of SDQ in the presence of detergents, the plaque-forming ability of the phage was assessed in various types of detergent. Briefly, SDQ was standardized to a titer of 10^8^ PFU/mL. Molten soft agar (5 mL, 0.75%) was mixed with 100 μL of SDQ and 100 μL of bacterial culture, and overlain on the surface of solidified basal LB agar. An SDQ suspension (10^8^ PFU/mL) was tested against *S. aureus* in the presence of detergents (SDS, CTAB, Tween 20, Triton X-100, CHAPS, or Brij 35) at concentrations of 1%, or with tap water, 10 mM EDTA, or 10% fetal bovine serum. The plaques were counted after incubation for 6–8 h at 37 °C. Each test was repeated three times. Established biofilms were also treated with SDQ, detergent, or a combination of phage (MOI = 10) + detergent. Biofilm formation was assessed at 0, 4, 8, 12, and 24 h. All the experiments were performed in triplicate.

### Statistical analysis

All data are presented as the means ± standard deviation (SD) of three or more independent experiments (*n* ≥ 3). One-way ANOVA followed by a *t* test (GraphPad Software Inc., San Diego, CA, USA) was used to evaluate differences between bacterial titers and between phage titers. Differences with *P* < 0.05 or *P* < 0.01 were considered significant (*) or highly significant (**), respectively.

## Supplementary Information

**Additional file 1: Table S1.** Storage stability of SDQ under refrigerated temperature.

**Additional file 2: Table S2.** List of S. aureus. Strains used in this study.

**Additional file 3: Table S3.** SDQ inhibited biofilm formation.

**Additional file 4.** Authors’ original data for Fig. 2

**Additional file 5.** Authors’ original data for Fig. 3.

**Additional file 6.** Authors’ original data for Fig. 4.

**Additional file 7.** Authors’ original data for Fig. 5.

**Additional file 8.** Authors’ original data for Fig. 7.

**Additional file 9.** Authors’ original data for Fig. 8.

**Additional file 10.** Authors’ original data for Fig. 9.

**Additional file 11.** Authors’ original data for Fig. 10.

## Data Availability

The datasets used and/or analysed during the current study are available from the corresponding author on reasonable request.

## Abbreviations

ATCC: American Type Culture Collection
CMCC: National Center for Medical Culture Collections
MRSA: methicillin-resistant *Staphylococcus aureus*
LB: Luria Bertani
TEM: Transmission electron microscopy
MOI: Multiplicity of infection
PFU: Plaque forming units
CFU: Colony-forming units
PBS: Phosphate buffer saline
SEM: Scanning electron microscopy
