# Supplementary material for: Potential of bacteriophages as disinfectants to control of Staphylococcus aureus biofilms
Source: BMC Microbiol. 2021 Feb 20;21:57. doi: 10.1186/s12866-021-02117-1 (PMC7896381; doi:10.1186/s12866-021-02117-1)
Supplement: Supplementary file 2 — Additional file 2: Table S2. List of S. aureus. Strains used in this study. [file 12866_2021_2117_MOESM2_ESM.docx]

**Table S2.** List of *S. aureus*. strains used in this study.

| **Isolate name** | **Sample number*** | **Specie of origin** | **MLST(ST)** | **Clonal Complex** | **Phage Sensitivity** |
| --- | --- | --- | --- | --- | --- |
| **SA 2Y3-4** | **2Y3-4** | **Bovine** | **97** | **CC97** | **+** |
| **SA 2Y9-1** | **2Y9-1** | **Bovine** | **97** | **CC97** | **+** |
| **SA 2Y7-1** | **2Y7-1** | **Bovine** | **5817** | **CC 97** | **+** |
| **SA 2Y4-3** | **2Y4-3** | **Bovine** | **239** | **CC 8** | **+** |
| **SA 7-1** | **7-1** | **Bovine** | **398** | **CC 398** | **-** |
| **SA 7-3** | **7-3** | **Bovine** | **398** | **CC 398** | **+** |
| **SA 8-1** | **8-1** | **Bovine** | **1** | **CC1** | **-** |
| **SA 8-2** | **8-2** | **Bovine** | **2154** | **CC 1** | **-** |
| **SA 8-3** | **8-3** | **Bovine** | **9** | **CC 1** | **-** |
| **SA 25-4** | **25-4** | **Bovine** | **239** | **CC 8** | **+** |
| **SA 25-5** | **25-5** | **Bovine** | **97** | **CC97** | **+** |
| **SA 27-2** | **27-2** | **Bovine** | **5796** | **CC 1** | **+** |
| **SA 4-2p** | **4-2p** | **Bovine** | **5** | **CC 5** | **+** |
| **SA-11-2-2p** | **11-2-2p** | **Bovine** | **398** | **CC 398** | **+** |
| **SA11-1** | **11-1** | **Bovine** | **97** | **CC 97** | **+** |
| **SA11-2** | **11-2** | **Bovine** | **97** | **CC 97** | **+** |
| **SA16b** | **16b** | **Bovine** | **398** | **CC 398** | **+** |

(+) = lytic; (-) = non lytic

*Isolate fields Country: Heilongjiang, China; Disease: mastitis; Source: milk.
